# Management of Spherophakia- or Microspherophakia-associated Mild to Moderate Glaucoma with Lensectomy, Anterior Vitrectomy, and Iris-Claw Intraocular Lens Implantation

**DOI:** 10.18502/jovr.v21.17682

**Published:** 2026-01-14

**Authors:** Aidin Meshksar, Masoumeh Masoumpour, Mohammad Hossein Nowroozzadeh, Reza Razeghinejad

**Affiliations:** ^1^Poostchi Ophthalmology Research Center, Department of Ophthalmology, School of Medicine, Shiraz University of Medical Sciences, Shiraz, Iran; ^2^Glaucoma Service, Wills Eye Hospital, Philadelphia, PA, USA

**Keywords:** Glaucoma, Iris claw lens, Lensectomy, Microspherophakia, Spherophakia

## Abstract

**Purpose:**

To report the outcomes of lensectomy, anterior vitrectomy, and iris-claw lens implantation in patients with spherophakia- or microspherophakia-related glaucoma.

**Methods:**

In this retrospective case series, we focused on patients with isolated microspherophakia or microspherophakia associated with various syndromes and mild to moderate angle-closure glaucoma who had undergone lensectomy, anterior vitrectomy, and iris-claw lens implantation.

**Results:**

We analyzed a total of 12 eyes of 6 patients with a mean age of 19 ± 6 years and a mean postoperative follow-up of 66 ± 12 months. All patients had lenticular myopia, and the mean refraction improved from - 7.26 ± 0.67 diopters (D) to - 1.18 ± 1.04 D after surgery. The mean corrected visual acuity improved from 0.92 ± 0.57 logMAR before surgery to 0.17 ± 0.15 logMAR at the last follow-up (*P* = 0.001). The mean intraocular pressure (IOP) decreased from 21.2 ± 3.9 mmHg on 1.9 ± 0.7 anti-glaucoma medications at baseline to 15.0 ± 1.5 mmHg (*P* = 0.006) on 0.8 ± 0.7 medications (*P* = 0.006) at the last follow-up.

**Conclusion:**

Lensectomy and iris-claw lens implantation in our cases not only decreased the IOP and reduced the number of glaucoma medications, but also improved the best-corrected visual acuity. Removal of the abnormally shaped lens likely contributed to these changes.

##  INTRODUCTION

In microspherophakia, the crystalline lens has a spherical shape with an increased anteroposterior diameter and a reduced equatorial diameter. The zonular fibers are elongated and weak. These changes result in excessive curvature of the anterior surface of the lens and a shallow anterior chamber.^[1]^ Microspherophakia and spherophakia may be isolated or associated with systemic disorders such as Weill-Marchesani syndrome, Marfan syndrome, homocystinuria, Klinefelter syndrome, and Alport syndrome. Visual loss in these conditions is secondary to high myopia, lens subluxation, or glaucoma. Glaucoma is the most common cause of permanent visual loss in this condition.

Several mechanisms have been proposed for the development of glaucoma in this condition, including pupillary block by a thick and unstable lens, peripheral anterior synechia, complete luxation of the lens into the anterior chamber, irritation of the ciliary body by the dislocated or subluxated lens, and congenital or acquired abnormality of the trabecular meshwork.^[2,3]^ The combination of laser peripheral iridotomy (LPI) and glaucoma medications may not control intraocular pressures (IOP), and surgical interventions such as trabeculectomy and tube-shunt implantation may be needed. Because of lens laxity and an inherent shallow anterior chamber, the risk of postoperative complications such as a flat anterior chamber and, consequently, cataract formation and corneal endothelial damage is high following filtering surgery.^[4]^ Therefore, lensectomy has been suggested for the management of spherophakia-associated glaucoma to address both glaucoma and the abnormal luxated lens.^[1]^ Depending on clinical and surgical conditions, the options for correcting aphakia include bag intraocular lens (IOL) implantation with capsular tension ring and fixation to the sclera, iris- or sclera-fixed IOL, anterior chamber IOL, or anterior chamber iris-claw IOL.^[5,6]^ Herein, we report the outcomes of lensectomy combined with anterior vitrectomy and iris-claw lens implantation in a series of patients with spherophakia or microspherophakia.

##  METHODS

This single-center, retrospective chart review study was conducted in patients with microspherophakia and mild-to-moderate angle-closure glaucoma. Diagnosis was based on visual field defects and/or characteristic optic disc abnormalities indicative of mild to moderate glaucoma. Patients with incomplete follow-up, advanced glaucoma, the need for combined lensectomy/iris-claw lens and glaucoma surgery, lens dislocation into the vitreous cavity, or a history of previous intraocular surgery—except for laser iridotomy—were excluded. Best-corrected visual acuity (BCVA), spherical equivalent, endothelial cell density, Goldmann applanation tonometry, and the number of glaucoma medications were compared before and after the surgical intervention.

### Surgical Technique

All surgeries were performed under general anesthesia by the same surgeon (RR). A 3.2 mm incision was created superiorly, and two side ports were placed nasally and temporally to the main incision. The anterior chamber was filled with an ophthalmic viscoelastic device (OVD) to maintain its** depth and protect corneal endothelial cells. Using a vitrectomy machine, the whole lens, including the capsule, was removed, and anterior vitrectomy was performed afterward. Next, 1% acetylcholine chloride intraocular solution was instilled into the anterior chamber, followed by OVD injection. After the main incision was extended, the Artisan IOL (Ophtec, Groningen, Netherlands) was inserted into the AC and centered on the pupil. With the help of the second instrument (a bent needle supplied by the lens manufacturer), the iris was enclavated into the haptics. Then, peripheral iridectomy was performed in patients with no history of laser iridotomy. The main incision was closed with three interrupted 10-0 nylon sutures, and the viscoelastic material was carefully removed. Postoperatively, all patients received a topical steroid and antibiotic drops every 6 hours. The antibiotic was stopped 10 days after the surgery, and the steroid was tapered and discontinued over a month. The anti-glaucoma medications were started based on the level of the postoperative IOP and the stage of glaucomatous optic neuropathy. Patients were followed postoperatively on day 1, week 1, month 4, and then every 3 to 6 months. During the follow-up period, loose nylon sutures were removed; the remaining sutures were removed by the end of the third post-operative month.

### Statistical Analysis

Quantitative and qualitative variables were described using mean ± standard deviation (SD) and frequency (percentage). The Wilcoxon signed-rank test was used to compare the preoperative and postoperative findings at the last follow-up. Each eye was treated as an independent observational unit. However, to account for intra-individual correlations arising from the inclusion of both eyes in some patients, we used Generalized Estimating Equations (GEE) with an unstructured working correlation matrix to obtain robust standard error estimates. Longitudinal changes in IOP over the follow-up period were evaluated using GEE modeling. Given the Poisson distribution of the number of anti-glaucoma medications and the small sample size, GEE was also applied for this outcome. In this regard, model fit was assessed using the Quasi-likelihood under the Independence Model Criterion (QIC) and its corrected version (QICC). The unstructured correlation matrix was chosen based on previously validated approaches.^[7,8]^ All statistical analyses were performed using SPSS version 25 (IBM Corp., Armonk, NY, USA), and a *P*-value < 0.05 was considered statistically significant.

**Figure 1:**
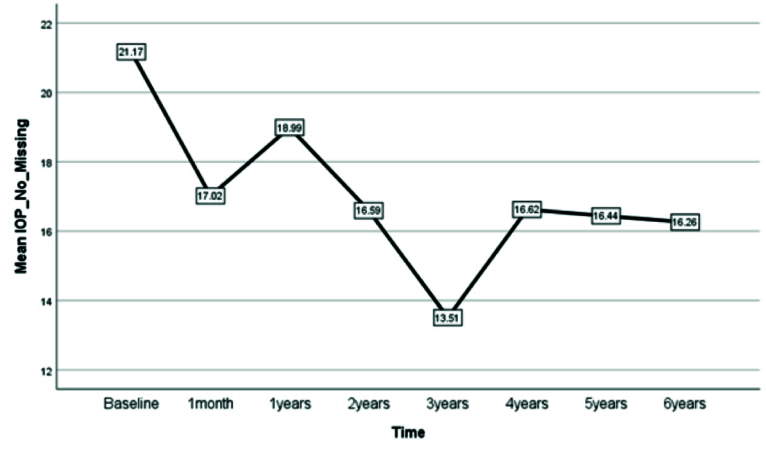
Postoperative intraocular pressure profile.

**Figure 2:**
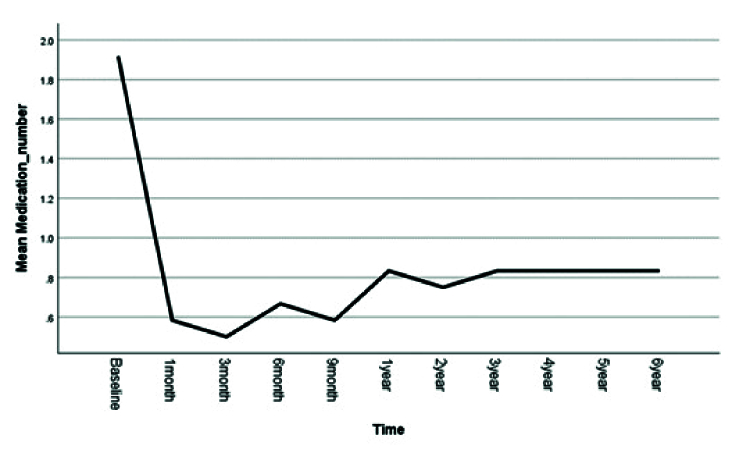
Pattern of the mean number of anti-glaucoma medications during the follow-up period.

**Table 1:** Demographic and clinical features of the patients

**Patient**	**Age at surgery (yr)**	**Sex**	**Diagnosis**	**Follow up (yr)**	**Eye**	**Pre-op BCVA (LogMar)**	**Final Post-op BCVA (LogMar)**	**Pre-op refraction**	**Post-op refraction**	**Pre-op IOP (mmHg)**	**Pre-op number of meds**	**Last visit IOP**	**Last visit number of medications**	**Central corneal thickness (µm)**	**WTW**	**Axial length (mm)**	**Pre-op specular microscopy (cells/mm^2^)**	**Post-op specular microscopy (cells/mm^2^)**	**Post-op complications**
1	24	M	WMS	4	OD	1.00	0.00	-6.75 -1.75 × 30	+0.75 -2.00 × 170	12	2	17	0	577	12	21.76	2234	1974	Choroidal effusion
					OS	2.08	0.00	-6.50 -2 × 15	+1.75 -3.00 × 150	19	2	14	0	577	12.2	21.54	2987	1776	
2	10	F	WMS	6	OD	1.18	0.00	-7.50 -1.25 × 170	-1.75 -1.00 × 20	24	2	18	0	596	12.8	22.04	3092	2869	Pupillary block due to LPI blockade
					OS	0.30	0.00	-7.50 -0.75 × 5	-0.25 -2.5 × 10	20	1	15	0	589	12.7	21.69	3221	3188	
3	24	F	Spherophakia	5	OD	0.52	0.04	-5.75 -2.5 × 120	0.00 -3.00 × 150	18	3	14	2	572	12.7	24.48	2234	2027	
					OS	0.52	0.00	-5.00 -2 × 170	+0.50 -1.00 × 20	21	3	14	2	575	12.4	24.45	2150	1850	
4	19	M	Spherophakia	6	OD	0.52	0.00	-7.00 -0.75 × 130	+0.50 -2.75 × 150	26	3	13	1	600	11.8	22.69	2769	2360	
					OS	1.30	0.00	-7.50 -1.25 × 115	-0.75 -1.75 × 160	20	2	16	1	583	11.9	22.49	2989	2433	
5	13	F	WMS	6	OD	0.39	0.00	-6.00 -1 × 106	-0.50 -0.75 × 100	26	2	20	1	613	12.2	19.48	2603	2405	
					OS	1.00	0.04	-6.75 -1.75 × 34	+1.00 -2.75 × 30	22	1	18	1	602	12.4	19.47	2531	2021	CME
6	25	F	WMS	4	OD	0.39	0.15	-5.50 -2.25 × 125	-1.75 -3.75 × 150	23	1	13	1	605	11.6	20.54	2991	2463	
					OS	0.52	0.15	-6.00 -1.75 × 170	Plano -2.75 × 21	23	1	14	1	595	11.5	20.81	2875	2755	
BCVA; best corrected visual acuity; UCVA, uncorrected visual acuity; CME, cystoid macular edema; LPI, laser peripheral iridotomy; M, male, F, female, WMS, Weill-Marchesani Syndrome, N/A, not available.																			

##  RESULTS

Between 2006 and 2016, 26 eyes of 14 patients with microspherophakia underwent surgery. Six patients with incomplete follow-up and two patients with severe glaucoma who received combined lensectomy, anterior vitrectomy, Artisan IOL implantation, and Ahmed glaucoma valve implantation were excluded. A total of 12 eyes from 6 patients (2 males and 4 females) who met the inclusion criteria were included. The demographic and clinical features of these patients are presented in Table 1. Four patients had Weill–Marchesani syndrome, and two patients had isolated microspherophakia. Seven eyes of four patients had undergone LPI before the surgical intervention. The mean ( ± SD) of the patients' age at presentation was 19 ± 6 years, ranging from 10 to 25 years. All patients were followed up for a minimum period of 4 years. The average follow-up was 66 ± 12 months (range, 48-84 months). The main visual complaint of all patients was decreased vision. Patients 1, 3, 4, 5, and 6 were referred due to elevated IOP and decreased vision. Patient 2 had an episode of acute IOP rise in the right eye. She had a flat anterior chamber nasally in both eyes, despite having patent peripheral iridotomies. The mean axial length, white-to-white diameter, and pachymetry were 21.80 ± 1.59 mm, 12.18 ± 0.43 mm, and 590.33 ± 13.46 microns, respectively. Only two eyes had Snellen BCVA of 20/40 preoperatively, and the others had worse visual acuity. Snellen BCVA of 10 eyes improved to 20/25 and greater after surgery. The logMAR (logarithm of the Minimum Angle of Resolution) BCVA improved from 0.92 ± 0.57 before surgery to 0.17 ± 0.15 at the last follow-up (*P* = 0.001). The mean IOP decreased from 23.87 ± 6.01 mmHg to 19.72 ± 6.47 mmHg in the first postoperative month (*P* < 0.001) [Figure 1]. No patient had an IOP > 22 mmHg at the last follow-up visit. The number of glaucoma medications decreased significantly from 1.9 ± 0.28 to 0.6 ± 0.18 in the first postoperative month (*P* = 0.001) and remained relatively stable for 6 years [Figure 2]. The mean spherical equivalent of refraction improved from - 7.26 ± 0.67 D to - 1.18 ± 1.04 D in the third postoperative month. The mean central endothelial cell density decreased significantly from 2723 ± 366.74 preoperatively to 2343.41 ± 436.02 in the last follow-up visit (13.95% reduction, *P* = 0.001). Regarding postoperative complications, patient 2 developed pupillary block and iris bombe in the right eye, despite having a patent laser iridotomy before surgery. The patient was unable to tolerate laser iridotomy due to the pain, high IOP, and poor cooperation (the patient was 6 years old); therefore, surgical iridectomy was performed. Patient 1 developed a non-kissing suprachoroidal effusion in the left eye secondary to corneal wound leakage on the second postoperative day and underwent suture revision and choroidal drainage. Patient 5 developed cystoid macular edema in the right eye. This was refractory to topical nonsteroidal anti-inflammatory agents and steroids, but it resolved completely with subtenon triamcinolone injection. Figure 1 shows the IOP profile of all patients during the follow-up period. The mean IOP at each time point was positively correlated with baseline IOP (*P* = 0.010) and negatively correlated with time (*P* = 0.001), demonstrating a non-linear association.

##  DISCUSSION

In this study, we found a significant decrease in IOP with lensectomy, anterior vitrectomy, and iris-claw lens implantation in eyes with microspherophakia. The IOP continuously decreased up to 3 years after surgery and slightly increased thereafter. Angle-closure glaucoma is a common finding in patients with microspherophakia and may result from a combination of progressive lens changes, anterior lens subluxation, pupillary block, peripheral anterior synechia formation, and irreversible damage to the trabecular meshwork.^[2]^ LPI is performed to relieve the pupillary block. Laser iridoplasty might be used after successful iridotomy to open the angle further.^[9]^ However, since the changes in lens shape and location are progressive, the angle progressively narrows over time.^[2,4,10]^ Anti-glaucoma medications effectively control IOP in only 18% of glaucomatous eyes with microspherophakia.^[11]^ The interventions for lowering IOP when laser and medical therapies fail include filtering procedures or lens extraction. Trabeculectomy may result in a flat anterior chamber due to the underlying anatomical problems of zonules. In a report, four eyes of two children with spherophakia developed flat anterior chamber after trabeculectomy, which did not respond to anterior chamber reformation and finally underwent lensectomy.^[4]^ However, Asaoka et al reported that trabeculectomy resulted in the successful management of one eye with glaucoma and spherophakia.^[12]^

The main objectives of surgical treatment in spherophakia are to reduce IOP and open the angle, but trabeculectomy may not effectively widen the narrow angle and prevent progressive PAS (peripheral anterior synechiae) formation. Trabeculectomy may further enhance PAS formation by increasing the chance of a shallow anterior chamber and postoperative inflammation. Separating the peripheral iris tissue from the trabecular meshwork at early stages of spherophakia-associated glaucoma may prevent the detrimental effect of iridotrabecular contact on the trabecular meshwork, which is achieved only by the extraction of the abnormally shaped lens. Additionally, trabeculectomy will not affect lenticular myopia and may even worsen it by further moving the lens forward. Generally, trabeculectomy accelerates cataract formation, with reports ranging from 15% to 60%,^[13]^ which necessitates a second procedure to address this complication.

In patients with spherophakia or microspherophakia, lensectomy is recommended if the lens is opacified, luxated into the anterior chamber or vitreous, or bisecting the pupil and associated with poor spectacle-corrected visual acuity.^[14]^ A multicenter, international randomized controlled clinical trial (the EAGLE study) showed that clear lens extraction in a patient with primary angle closure and primary angle closure glaucoma lowered the IOP and was more cost-effective than LPI and antiglaucoma medications; however, the study did not establish the role of lens extraction in the management of spherophakic glaucoma.^[15]^ Yaser reported a case that had a good IOP response to lensectomy for 2 years, but had to undergo trabeculectomy due to uncontrolled IOP in both eyes.^[16]^ Kanamori et al^[17]^ reported good IOP control following goniosynechiolysis with lens aspiration and IOL implantation in a patient with spherophakia and chronic angle closure glaucoma. Another study reported a case of Weill-Marchesani syndrome with microspherophakia that was managed successfully with pars plana lensectomy combined with goniotomy.^[18]^

Surgical correction of aphakia without capsular support remains a challenge. Besides prescribing glasses or contact lenses and leaving the patient aphakic, current surgical options include anterior chamber IOL implantation, iris- or scleral-sutured IOLs, iris-claw IOLs, and the use of capsular tension rings/segments with intrabag IOL implantation.^[14,19,20,21]^ Although angle-supported anterior chamber IOL implantation is technically easier, it has been associated with several complications related to the iridocorneal angle, IOP elevation, and corneal endothelial cell loss.^[22]^ Additionally, placing an anterior chamber IOL haptic in eyes with already compromised trabecular meshwork may cause more trabecular damage. Anterior chamber IOLs are claimed to be associated with a higher incidence of uveitis-glaucoma-hyphema syndrome than other IOL implantation methods.^[23]^ Transscleral fixation of posterior chamber IOLs is a technically demanding procedure with a relatively higher risk of intra- and postoperative complications. It can be performed either as sutureless or sutured scleral fixation.

Although the long-term outcomes of scleral-fixated IOLs in children are unknown, several studies have demonstrated encouraging short-term results. Meanwhile, it should be stressed that scleral-fixated IOLs need effective vitrectomy and proper scleral tunnel fixation, and children have a less rigid sclera compared to adults. Also, suture breakage, hyphema, vitreous hemorrhage, cystoid macular edema, and retinal detachment are the reported complications of scleral-fixated IOLs. Additionally, scleral-fixated IOLs and anterior chamber IOLs cannot be used in patients with a white-to-white diameter of > 13 mm, and iris-claw lenses are preferred in these cases due to the possibility of IOL decentration or dislocation.^[24,25,26,27]^

In our series, BCVA was greater postoperatively, although the patients did not have cataracts. This could be attributed to eliminating lens-induced astigmatism and aberrations after surgery. Also, our patients generally had better visual acuity outcomes than those in the study by Khokhar, who implanted anterior chamber IOLs or scleral-fixated IOLs, or left patients aphakic.^[27]^ In our series, IOP decreased significantly after surgery, and patients' dependence on medications was reduced. Once the trabecular outflow is damaged, IOP may remain elevated despite opening the angle following lens extraction. Requiring additional anti-glaucoma medications suggests that lens removal alone may not sufficiently lower IOP in some patients with synechial angle closure, even after synechiolysis.

In our series, the patients did not face sight-threatening complications. Macular edema occurred in one eye and resolved completely after a subtenon injection of triamcinolone. The suprachoroidal effusion was due to corneal wound leakage, which resolved with wound revision and choroidal drainage. The patient who developed pupillary block despite prior LPI responded well to surgical iridectomy. Artisan aphakic IOL may be associated with uveitis-glaucoma-hyphema syndrome, lens dislocation, corneal decompensation, retinal detachment, and pupil distortion;^[28]^ however, we did not observe any of these complications. In another study by Forlini et al, 320 eyes of 320 adult patients without capsular support underwent retro-pupillary iris-claw lens implantation and were followed up for a mean duration of 5.3 years. The reported complications included disenclavation of the IOL (three cases), dislocation into the vitreous (one case), retinal detachment (one case), chronic dull pain (eight cases), and macular edema (one case). There was no statistically significant change in the endothelial cell density at the end of the follow-up period. Furthermore, Forlini et al observed IOP elevation in seven cases, but they were managed medically.^[21]^

The present study has several limitations, including its small sample size and retrospective design. Given the rarity of spherophakia- or microspherophakia-related glaucoma, conducting a prospective study with an acceptable sample size does not seem feasible. In summary, in our series, lensectomy combined with anterior vitrectomy and iris-claw lens implantation in spherophakia-associated glaucoma resulted in improved vision and decreased IOP; however, the aforementioned limitations can significantly affect the generalizability of these results.

##  Financial Support and Sponsorship

None.

##  Conflicts of Interest

None.

## References

[B1] Hatt SR, Gnanaraj L (2013). Interventions for intermittent exotropia. Cochrane Database Syst Rev.

[B2] Chia A, Roy L, Seenyen L (2007). Comitant horizontal strabismus: An Asian perspective. Br J Ophthalmol.

[B3] Chia A, Seenyen L, Long QB (2005). A retrospective review of 287 consecutive children in Singapore presenting with intermittent exotropia. J AAPOS.

[B4] Govindan M, Mohney BG, Diehl NN, Burke JP (2005). Incidence and types of childhood exotropia: A population-based study. Ophthalmology.

[B5] Jenkins RH (1992). Demographics: Geographic variations in the prevalence and management of exotropia. Am Orthopt J.

[B6] Khorrami-Nejad M, Akbari MR, Khosravi B (2018). The prevalence of strabismus types in strabismic Iranian patients. Clin Optom.

[B7] Mohney BG, Huffaker RK (2003). Common forms of childhood exotropia. Ophthalmology.

[B8] Burian HM (1966). Exodeviations: Their classification, diagnosis and treatment. Am J Ophthalmol.

[B9] Shen PC, Zhang Y, Liu Y, Jiang J, Xu JJ, Lin HL (2018). Study on the difference of binocular accommodative response between patients with intermittent exotropia under different viewing condition. Zhonghua Yan Ke Za Zhi.

[B10] Burian HM, Spivey BE (1965). The surgical management of exodeviations. Am J Ophthalmol.

[B11] Kushner BJ (1988). Exotropic deviations: A functional classification and approach to treatment. Am Orthopt J.

[B12] Kushner BJ, Morton GV (1998). Distance/near differences in intermittent exotropia. Arch Ophthalmol.

[B13] Lavrich JB (2015). Intermittent exotropia: Continued controversies and current management. Curr Opin Ophthalmol.

[B14] Romano PE, Wilson MF, Campos EC (1990). Survey of current management of intermittent exotropia in the USA and Canada. Strabismus and ocular motility disorders.

[B15] Akbari MR, Mirzajani A, Moeinitabar MR, Mirmohammadsadeghi A, Khorrami-Nejad M, Sharbatoghli L (2019). The effect of alternate occlusion on control of intermittent exotropia in children. Eur J Ophthalmol.

[B16] Davies LN, Mallen EA, Wolffsohn JS, Gilmartin B (2003). Clinical evaluation of the shin-nippon NVision-K 5001/grand seiko WR-5100K autorefractor. Optom Vis Sci.

[B17] Ahn SJ, Yang HK, Hwang J-M (2012). Binocular visual acuity in intermittent exotropia: Role of accommodative convergence. Am J Ophthalmol.

[B18] Ha SG, Jang SM, Cho YA, Kim SH, Song JS, Suh YW (2016). Clinical exhibition of increased accommodative loads for binocular fusion in patients with basic intermittent exotropia. BMC Ophthalmol.

[B19] Somer D, Demirci S, Çınar FG, Duman S (2007). Accommodative ability in exotropia: Predictive value of surgical success. J AAPOS.

[B20] Mohney B, Holmes J, de Faber J-THN (1988). Classification of control in intermittent exotropia. Transactions 28th European Strabismological Association Meeting.

[B21] Mohney BG, Holmes JM (2006). An office-based scale for assessing control in intermittent exotropia. Strabismus.

[B22] Stathacopoulos RA, Rosenbaum AL, Zanoni D, Stager DR, McCall LC, Ziffer AJ (1993). Distance stereoacuity. Assessing control in intermittent exotropia. Ophthalmology.

[B23] Sharma P, Saxena R, Narvekar M, Gadia R, Menon V (2008). Evaluation of distance and near stereoacuity and fusional vergence in intermittent exotropia. Indian J Ophthalmol.

[B24] O’Neal TD, Rosenbaum AL, Stathacopoulos RA (1995). Distance stereo acuity improvement in intermittent exotropic patients following strabismus surgery. J Pediatr Ophthalmol Strabismus.

[B25] Gökgöz Özişik G, Gokgoz G, Caglar C, Cakmak HB (2022). Relationship between the clinical factors and deviation control in intermittent exotropia. J Pediatr Ophthalmol Strabismus.

[B26] Superstein R, Dean TW, Holmes JM, Chandler DL, Cotter SA, Wallace DK, Pediatric Eye Disease Investigator Group (2017). Relationship among clinical factors in childhood intermittent exotropia. J AAPOS.

[B27] Kang KT, Lee SY (2015). Relationship between control grade, stereoacuity and surgical success in basic intermittent exotropia. Korean J Ophthalmol.

[28] Lett KS, Chaudhuri PR (2011). Visual outcomes following Artisan aphakia iris claw lens implantation. Eye.

